# The bowel and beyond: extracolonic findings from CT colonography

**DOI:** 10.1007/s11845-021-02595-2

**Published:** 2021-03-24

**Authors:** Gerard Lambe, Peter Hughes, Louise Rice, Caoimhe McDonnell, Mark Murphy, Ciaran Judge, Michael Guiney

**Affiliations:** 1 Radiology Department, St. James’s Hospital, James’s Street, Dublin 8, Ireland; 2 Radiology Department, The Mater Misericordiae University Hospital, Eccles Street, Dublin 7, Ireland; 3 Gastroenterology Department, St. James’s Hospital, James’s Street, Dublin 8, Ireland

**Keywords:** C-RADS, CT colonography, Extracolonic findings, Screening, Symptomatic

## Abstract

CT colonography has emerged as the investigation of choice for suspected colorectal cancer in patients when a colonoscopy in incomplete, is deemed high risk or is declined because of patient preference. Unlike a traditional colonoscopy, it frequently reveals extracolonic as well as colonic findings. Our study aimed to determine the prevalence, characteristics and potential significance of extracolonic findings on CT colonography within our own institution. A retrospective review was performed of 502 patients who underwent CT colonography in our institution between January 1, 2010 and January 4, 2015. Of 502 patients, 60.63% had at least one extracolonic finding. This was close to other similar-sized studies (Kumar et al. Radiology 236(2):519–526, 2005). However, our rate of E4 findings was significantly higher than that reported in larger studies at 5.3%(Pooler et al. AJR 206:313–318, 2016). The difference may be explained by our combination of symptomatic/screening patients or by the age and gender distribution of our population. Our study lends support to the hypothesis that CT colonography may be particularly useful in identifying clinically significant extracolonic findings in symptomatic patients. CT colonography may allow early identification of extracolonic malignancies and life-threatening conditions such as an abdominal aortic aneurysm at a preclinical stage when they are amenable to medical or surgical intervention. However, extracolonic findings may also result in unnecessary investigations for subsequently benign findings.

## Introduction

Colonoscopy is the first-line investigation for suspected colorectal cancer. For those patients in whom a colonoscopy is incomplete, is deemed high risk or is declined because of patient preference, CT colonography (CTC) is the next investigation of choice.

CT colonography has several advantages over a traditional colonoscopy. It allows a complete examination of the abdomen and pelvis. It is a relatively safe investigation that is well tolerated by most patients. It was accepted as a screening tool for colorectal cancer by the American Cancer Society in 2008. Importantly, it also raises the possibility of uncovering extracolonic findings which remain blind to endoscopic examination. Radiologists must report both colonic and extracolonic findings.

A systematic review by Xiong et al. found that 40% of patients undergoing CT colonography had at least one extracolonic finding. Fourteen percent of all patients had a “significant finding” requiring further investigation [1]. Pooler et al. looked at E4 (potentially significant) findings in a screening population and found that 2.5% had E4 findings [2].

The aim of our study was to determine the prevalence and characteristics of extracolonic findings from CT colonography within our own institution.

## Methods

A retrospective analysis was conducted of all CT colonography studies performed in our institution from January 1, 2010 to January 4, 2015 using the picture archiving and communication system (PACS). All studies in this period were included in this analysis regardless of the underlying indication for the scan. The total number of patients who underwent CT colonography during the study period was 502. This comprised 350 females and 152 males with a mean age of 66.63 years.

Patients were administered 20 mg of intravenous hyoscine butylbromide before the scan. They were asked to position themselves in a lateral decubitus position, and a catheter tip was introduced to the rectum. The colon was insufflated with four litres of carbon dioxide to a pressure of 20–25 psi. A topogram was performed before the supine scan to ensure adequate colonic distension. Additional carbon dioxide was administered before the prone scan if tolerated by the patient. No intravenous contrast was administered.

Examinations were performed on a 64-slice Toshiba scanner using a slice collimation of 5 mm, a pitch of 0.8 and a kVp of 120. Images were networked to a workstation using customised software. All CTs were read by one of two consultant radiologists, each with over 10 years of professional experience.

The formal reports of these studies were examined, and any extracolonic findings were identified. These findings were classified according to the CT colonography reporting and data system [3] (C-RADS) as E0, E1, E2, E3 or E4 (Table 1).

## Results

In total, 303 of 502 patients (60.36%) had at least one extracolonic finding. This included 27 patients (5.3%) who had an E4 finding (Table 2).

**Fig. 1:**
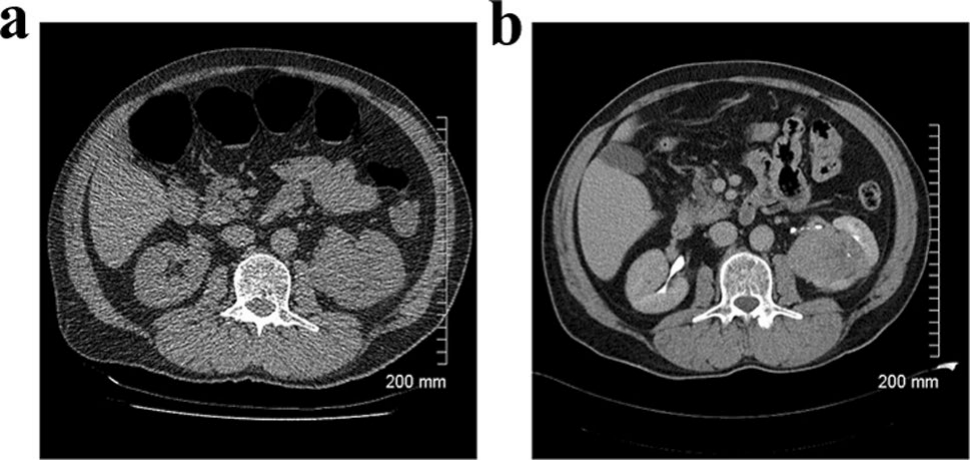
**a **Axial non-contrast CT shows a soft tissue mass arising from the interpolar region of the left kidney. **b** Axial contrast-enhanced CT confirms a 6.4-cm mass. Subsequent histopathology is consistent with a renal cell carcinoma

**Fig. 2:**
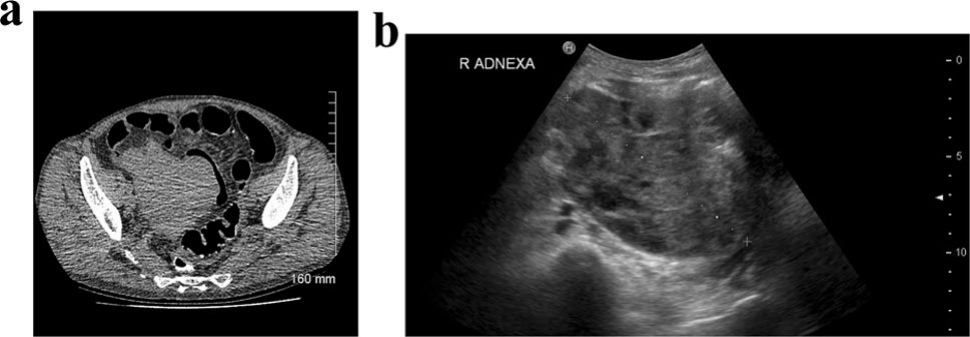
**a** Axial non-contrast CT shows a large soft tissue mass in the right pelvis. **b **Subsequent pelvic ultrasound confirms a mixed solid/cystic mass arising from the right ovary. Histopathology is consistent with an ovarian carcinoma

**Fig. 3:**
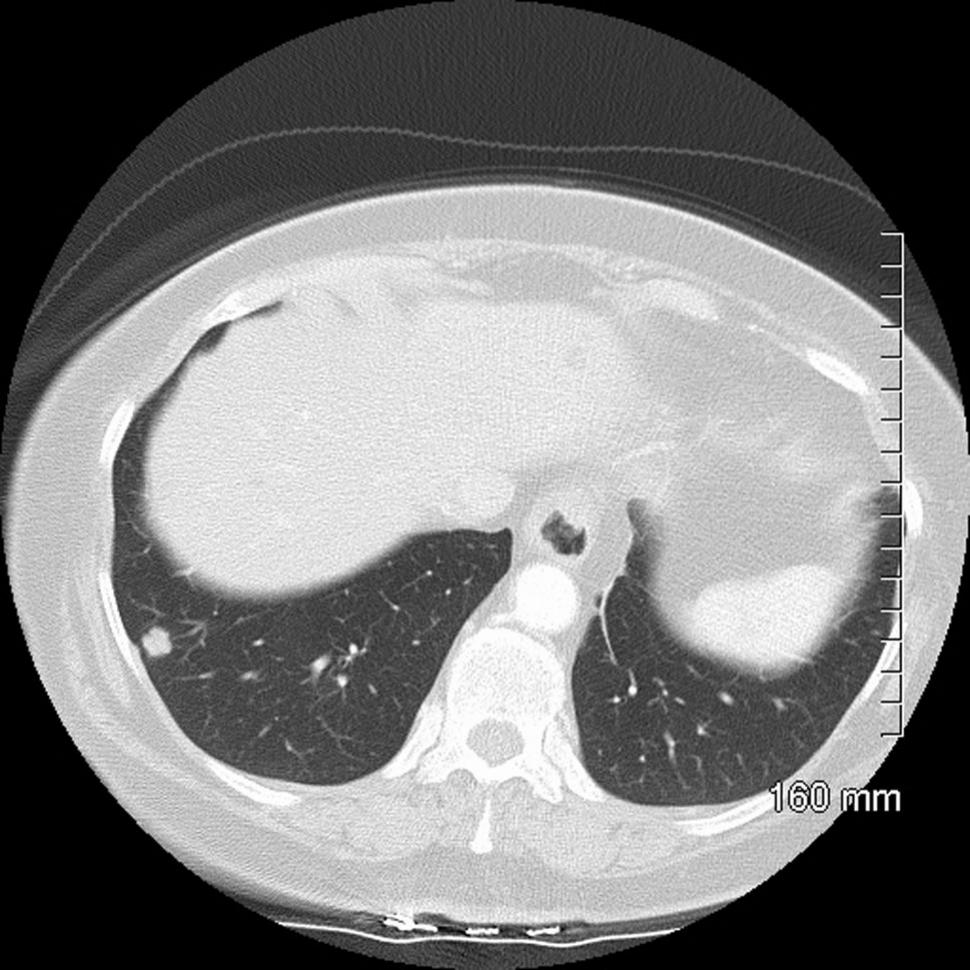
Axial non-contrast CT shows a 9-mm nodule in the right lower lobe. Subsequent histopathology is consistent with an atypical carcinoid tumour

The most common benign (E2) findings were renal cyst (*n* = 54), gallbladder calculus (*n* = 38), hiatus hernia (*n* = 34), renal calculus (*n *= 25) and atherosclerotic aorta (*n* = 24) (Table 3 ). The most common benign but important (E3/4) findings were pulmonary nodule (*n* = 14), renal mass (*n* = 11), complex liver lesion (*n* = 7), lymphadenopathy (*n* = 4) and adrenal mass (*n* = 3). Three extracolonic malignancies were incidentally identified on CT colonography during the study period—a renal cell carcinoma (Fig. 1a, b), an ovarian carcinoma (Fig. 2a, b) and a lung carcinoid (Fig. 3), all of which were surgically resected.

## Discussion

Our study aimed to determine the prevalence, characteristics and potential significance of extracolonic findings on CT colonography within our own institution and to compare our experience with that of other centres.

A retrospective review by Hara et al. first drew attention to extracolonic findings from CT colonography [4]. They found that 30 (11%) of 264 patients at high risk of colorectal cancer had highly important extracolonic findings. Pickhardt et al. found that extracolonic cancer exceeded colorectal cancer in a retrospective review of 10,286 patients in a CT colonography screening population [5]. However, a 2009 commentary predicted a “deluge” of incidental findings from CT colonographies which would drive up costs, anxiety, morbidity and mortality [6].

The major advantage of CT colonography is the early identification of extracolonic malignancies and life-threatening conditions such as an abdominal aortic aneurysm at a preclinical stage when they are still amenable to medical or surgical intervention. It is a relatively safe investigation which is well tolerated by the majority of patients.

One disadvantage of CT colonography is radiation exposure in a situation where the alternative (optical colonoscopy) is radiation-free. Modern CT scanners with a low-dosage protocol can keep doses under 5 mSv. However, an increased risk of malignancy seems likely, particularly for older patients and those undergoing multiple scans [7]. There is significant expense associated with further investigations. One study found that the mean cost of working up unexpected findings from CT colonography was approximately equal to the cost of the CT colonography itself [8]. Halligan et al. found that the average cost per patient of working up extracolonic findings was £99 v £5 for CT colonography v barium enema, and £153 v £0 for CT colonography v colonoscopy [9].

In addition, extracolonic findings may result in “unnecessary” investigations for subsequently benign findings. However, Plumb et al. used a discreet choice experiment in a CT colonography screening population to establish that both patients and healthcare professionals believe that the diagnosis of an extracolonic malignancy from a screening CT colonography outweighs the potential disadvantages of further imaging and invasive investigations brought about by false positive results. For patients, the median tipping point was 99.8% for radiological follow-up and 10% for invasive follow-up. The median tipping point for healthcare professionals was 40% for radiological follow-up and 5% for invasive follow-up. It appears that the specificity of a CT colonography in a screening population is likely to be highly acceptable to both patients and doctors [10].

The reported incidence of extracolonic findings in the literature is variable. The overall incidence in our study was 60.63%. This was close to other similar-sized studies [11]. Smaller studies have reported lower incidences. The incidence was 41% in a study by Hara et al. [4] and 15% in a study by Edwards et al. [12]. However, our rate of E4 findings was significantly higher than that reported in the largest study of 7952 screening patients [2].

It was suggested that this may be due to our own combination of symptomatic and screening patients. However, a recent 2019 study of 388 patients found no statistically significant difference in *E* scores or clinical outcomes of extracolonic findings between symptomatic and screening patients [20]. It is likely that other factors such as the age and gender distribution of our population are at play.

An increased incidence of extracolonic findings from CT colonography is associated with the use of intravenous contrast [13], high-dosage radiation protocols [14], increasing age [15] and female gender [16]. In particular, the predominance of females in our study most likely explains the high number of E3/E4 findings, since other studies have found that up to 25% of E3 findings are adnexal or uterine lesions [21].

Symptomatic patients with a colonic finding are less likely to have an extracolonic finding, while symptomatic patients without a colonic finding are more likely to have an extracolonic finding investigated [16]. In symptomatic patients, it is thought that up to 10% of extracolonic findings may account for the patient’s symptoms from their initial presentation [17].

The CT colonography reporting and data system establishes a standard approach to the reporting of colonic and extracolonic findings and acts as a guide to management by estimating the clinical significance of these findings. A study of 2277 screening patients found that 46% had at least one extracolonic finding, but only 11% were E3/4 [19]. A study of symptomatic patients found double the rate of E3/4 findings [9]. Our own study population was a combination of screening and symptomatic patients, and the rate of E3/4 findings was 18%. This lends support to the conclusion that CT colonography may be particularly useful in symptomatic patients.

Our study identified three extracolonic malignancies from 502 patients. It could be hypothesised that CT colonography accelerated these diagnoses. One prospective, randomised trial of patients with symptoms of colorectal cancer found that extracolonic cancer was indeed diagnosed at twice the expected rate for the general population at 1 year post randomisation to CT colonography, but time to diagnosis was not reduced compared with patients who underwent a barium enema or colonoscopy [9]. This would suggest that patients who underwent a barium enema or colonoscopy as their initial investigation may have undergone further abdominopelvic imaging as a result of persistent abdominal symptoms not explained by the initial test, ultimately leading to a diagnosis of extracolonic cancer in a similar timeframe to patients who had a CT colonography up front.

It has been suggested that radiographers may have a role in identifying extracolonic findings at the time of the scan and performing further same-day imaging if and when required. However, a Dutch study in 2012 invited eight radiographers to engage in a structured training programme, to triage cases based on the CT colonography reporting and data system and to flag the appropriate scans for a radiologist review. They found that correct identification of E3 findings improved from 52 to 70% after training, but identification of E4 findings was unchanged at 69% [18]. As such, radiographers should not be expected to identify all extracolonic findings.

Our study was limited to a single centre. The CT colonography reporting and data system was designed for screening rather than symptomatic investigations, and the absence of a comprehensive classification table means that the *E* score given is dependent on the subjective opinion of the reporting radiologist. Two radiologists report CT colonography studies in our institution, and their personal thresholds for reporting extracolonic findings may vary, particularly for those that are perceived to be low risk.

The National Bowel Screening Programme, Bowel Screen, was rolled out in 2012. While CT colonography studies have largely been deferred in our institution since the start of the COVID-19 pandemic, they have recently been restarted on a regular basis. The resultant backlog is likely to result in greater pressure on CT colonography services at a local and national level.

In summary, our study aimed to assess the prevalence, characteristics and potential significance of extracolonic findings on CT colonography within our own institution. We found a similar rate of extracolonic findings to other similar-sized studies [11] but a higher rate of E4 findings than larger studies [2]. Our study lends support to the hypothesis that CT colonography may be particularly useful in identifying clinically significant extracolonic findings in symptomatic patients, and this will bring both opportunities and challenges in the years ahead.

**Table 1:** Summary of CT colonography reporting and data system colorectal and extracolonic classification scores

Score	Description
Colorectal	
C0, inadequate study	Inadequate preparation; inadequate insufflation
Cl, normal colon or benign lesion	No polyp [greater than or equal to] 6 mm; recommend routine screening with CT colonography or colonoscopy in 5 years
C2, intermediate polyp or indeterminate finding	Polyps 6–9 mm,< 3 in number; recommend CT colonography polyp surveillance or colonoscopy with polypectomy
C3, polyp, possibly advanced adenoma	Polyps [greater than or equal to] 10 mm; [greater than or equal to] 3 polyps, each 6–9 mm; recommend colonoscopy with polypectomy
C4, colorectal mass, like ly malignant	Lesion compromises bowel lumen, shows extracolonic invasion; recommend surgical consultation
Extracolonic	
E0, limited examination	Compromised by artifact; evaluation of extra colonic tissues severely limited; not used in practice by our program
El, normal examination or anatomic variant	No extracolonic abnormalities visible; no workup indicated
E2, clinically unimportant finding	Examples: simple liver or kidney cyst, cholelithiasis without cholecystitis; no workup indicated
E3, likely unimportant, incompletely characterised	Example: minimally complex or homogeneously hyperattenuating kidney cyst; workup may be indicated; dependent on specific clinical scenario
E4, potentially important finding	Examples: solid kidney mass, aortic aneurysm; workup generally indicated, but dependent on specific clinical scenario; communicate to referring physician as per accepted practice guidelines

Table is based on data published elsewhere [41]

**Table 2:** The number of E0, E1, E2, E3 and E4 findings in our study group

C-RADS *E* score	Total CT colonography (*n* = 502)
EO	5
El	194
E2	212
E3	61
E4	30

**Table 3:** The relative number of benign and malignant extracolonic findings are illustrated

Benign finding	Number	Malignant finding	Number
Renal cyst	54	Ovarian carcinoma	1
Gallbladder calculus	38	Renal cell carcinoma	1
Hiatus hernia	34	Lung carcinoid	1
Renal calculus	25	Total	3
Atherosclerotic aorta	24		
Hepatic cyst	22		
Abdominal/pelvic hernia	18		
Granulomatous disease	16		
Pulmonary nodule	14		
Abdominal aortic aneursym <S cm	13		
Emphysema	12		
Renal mass	11		
Adrenal adenoma/hyperplasia	10		
Fatty liver	8		
Renal scarring/atrophy	7		
Complex liver lesion	7		
Vertebral fracture	7		
Adnexal cyst	6		
Abdominal/pelvic lymphadenopathy	4		
Portal HTN/Chronic liver disease	3		
Uterine fibroids	3		
Pectus excavatum/carinatum	3		
Spinal scolioisis	3		
Adnexal mass	3		
Bronchiectasis	3		
Adrenal mass	3		
Splenic cyst	3		
Prostatomega ly	2		
Chronic interstitial lung disease	2		
Splenunculus	2		
Chronic pancreatitis	2		
Pleural plaques	2		
Splenomegaly	2		
Horseshoe kidney	2		
Spondylosis	2		
Complex bladder lesion	2		
Pulmonary groundglass attenuation	2		
Complex pancreatic lesion	2		
Angiomyolipoma	2		
Peritoneal deposits	1		
Obstructing renal calculus	1		
Breast mass	1		
Gallbladder thickening	1		
Groin mass	1		
Pancreatic cyst	1		
Ectasia of the abdominal aorta	1		
Calcified lymph nodes	1		
Sacroiliitis	1		
Sacral meningocele	1		
Absent kidney	1		
Pars defect	1		
Adrenal haemorrhage	1		
Hepatomegaly	1		
Dilated common bile duct	1		
Bladder calculus	1		
IPMN lesion of the pancreas	1		
Liver haemangioma	1		
Common iliac aneurysm	1		
Fusion of L2 and L3	1		
Spondylolisthesis	1		
Thoracic aortic aneurysm	1		
Total	400		
